# Prevalence of Apical Periodontitis and Its Association With Missed Canals and the Quality of Endodontic Filling Using Cone-Beam Computed Tomography (CBCT)

**DOI:** 10.7759/cureus.113444

**Published:** 2026-07-27

**Authors:** Fabien Fayez Jarjour, Yara Haidar, Jad Sabbagh, Ghenwa Touma, Antonio Oueiny, Ihab Ghoussainy, Alexandre Khairallah

**Affiliations:** 1 Faculty of Dentistry, Lebanese University, Beirut, LBN; 2 Department of Prosthodontics, Saint Joseph University of Beirut, Beirut, LBN; 3 Department of Maxillofacial Imaging, Lebanese University, Beirut, LBN

**Keywords:** apical periodontitis, cbct, endodontic, filling, missed canal, periapical lesion, teeth

## Abstract

Introduction: The success of endodontic treatment is mostly determined by the absence of apical periodontitis. This cross-sectional study aims to assess the prevalence of apical periodontitis and its association with missed canals and the quality of endodontic filling using cone-beam computed tomography (CBCT) in a Lebanese subpopulation.

Methods: One hundred and eighty-three CBCTs were analyzed by a 30-year experienced oral and maxillofacial radiologist over the period of three months. Every case of apical periodontitis was identified, and in every endodontically treated tooth, the quality of endodontic filling was recorded along with the presence of missed canals. All statistical analyses were performed using IBM SPSS Statistics for Windows, Version 26.0 (IBM Corp., Armonk, New York, United States). A significance level of α=0.05 was applied to all analyses.

Results: Four hundred and twenty-four cases of periapical periodontitis in endodontically treated teeth were identified. Forty-five (5.1%) of endodontically treated teeth presented with a missed canal, with 16 (35.56%) being the second mesiobuccal canal. Out of those, 24 (53.3%) showed a periapical lesion formation. Most canals, 584 (66.7%), presented with an adequate root canal filling. However, the overfilled ones showed a high prevalence of periapical lesions, seven (58.3%), followed by 139 (49.8%) in short-filled canals.

Conclusion: This study found a high prevalence of periapical lesions in endodontically treated teeth, particularly in teeth with missed canals and short and overfilled canals.

## Introduction

Apical periodontitis is an inflammatory condition affecting the periapical tissues, triggered by bacterial infection that elicits the host's immune response against the microorganisms and their by-products [1]. Without dental intervention, the infection may persist and extend to nearby periradicular tissues [1]. Apical periodontitis is considered globally as one of the most prevalent infectious oral diseases [2-4]. Multiple epidemiological surveys from Europe, North America, and Australia indicated that apical periodontitis is particularly common in adults, with prevalence estimates ranging between 27% and 70% [5]. Although apical periodontitis commonly arises from dental caries [6], multiple studies indicated a higher prevalence of apical periodontitis in endodontically treated teeth (ETT) [7].

Endodontic therapy aims to eliminate or markedly reduce the microbial load within the root canal system through comprehensive chemo-mechanical debridement followed by three-dimensional obturation [8]. Successful root canal treatment requires thorough canal shaping, effective disinfection, and complete filling of the root canal system; failure in any of these steps is closely linked to treatment failure [9]. Although root canal treatment is widely performed, apical periodontitis continues to be frequently observed [7,10]. Numerous studies determine that poor-quality endodontic procedures present a major etiological factor in apical periodontitis, emphasizing the importance of high technical principles during root canal treatment to maintain periradicular tissue health [5].

Adequate anatomical knowledge of the root canal system is essential for predictable endodontic success [11]. Undetected canals during root canal treatment, as they harbor microbial niches that perpetuate periapical pathology, significantly compromise treatment outcomes, contributing to the need for retreatment procedures [9,11,12]. Evidence indicates 98% of teeth with untreated canals develop apical periodontitis, making it among one of the principal risk factors [9,10].

Apical extent of root canal filling represents a major determinant of treatment quality [13,14]. Some studies demonstrate that fillings terminating 0-2 mm from the radiographical apex achieve significantly higher success rates than short (>2 mm) or overfilled canals, though other research contradicts this. Debate persists regarding optimal obturation length as it was seen at all obturation lengths [13]. Short fillings result from inadequate instrumentation, indicating insufficient cleaning with higher bacterial populations. Over-instrumentation causes material extrusion including infected debris and dentin particles that irritate periapical tissues [13,14]. Radiographic voids correlate with less favorable outcomes as they represent residual tissue or debris suggesting inadequate debridement and ongoing infection or technical obturation complications; however, some studies report filling homogeneity has no significant outcome influence [13,15].

In most old studies, periapical radiographs and orthopantomography have been the primary tools for the diagnosis of the presence of apical periodontitis [10]. Their known limitations, being two-dimensional images and the inability to detect missed untreated canals, led to a drawback in their usage [9,10]. With the development of three-dimensional imaging techniques, recent studies are utilizing cone-beam computed tomography (CBCT) [9,10]. CBCT, with its higher resolution, presents superior detection of periapical periodontitis while identifying the underlying causes, including missed canals, with its precise localization and greater sensitivity in evaluating root canal treatment quality [9-11].

Several studies have been conducted using CBCT to evaluate the relationship between the presence of apical periodontitis in ETT and the presence of missed canals and with that of the length/quality of the root canal treatment [10]. This cross-sectional study aimed to assess, using CBCT, the prevalence of apical periodontitis in a Lebanese subpopulation and to evaluate its association with endodontic treatment status, missed canals, and root canal filling length/quality.

## Materials and methods

A total of 183 CBCT images were collected from the Department of Radiology in the Faculty of Dentistry, Lebanese University, Beirut, Lebanon. These were originally recorded between January 2023 and January 2025, using a CS 9600 system (Carestream Dental LLC, Atlanta, Georgia, United States) for diagnostic or treatment purposes. Scans were acquired using standardized exposure parameters of 90 kV, 8 mA, and a medium field of view (FOV) of 10×10 cm. Every scan was studied in all viewing planes to ensure that no missing data was overlooked. Every patient over 18 years old provided written informed consent permitting the anonymous use of their scans for future research activities.

The inclusion criteria included every permanent tooth with fully completed roots and those that were endodontically treated should have been completed more than one year prior. Every CBCT image of poor quality or showing artifacts was excluded, along with patients taking systemic medications and whose teeth had undergone previous endodontic surgery, root fractures, root perforations, or external root resorption.

The 183 CBCT images, divided between 85 males and 98 females, all met the inclusion criteria. They were studied by a 30-year experienced oral and maxillofacial radiologist over the period of three months. To ensure reliability and limit observer bias, the evaluations were repeated after a six-month interval without access to the initial assessment. Discrepant findings were addressed by using the mean values for analysis.

**Figure 1:**
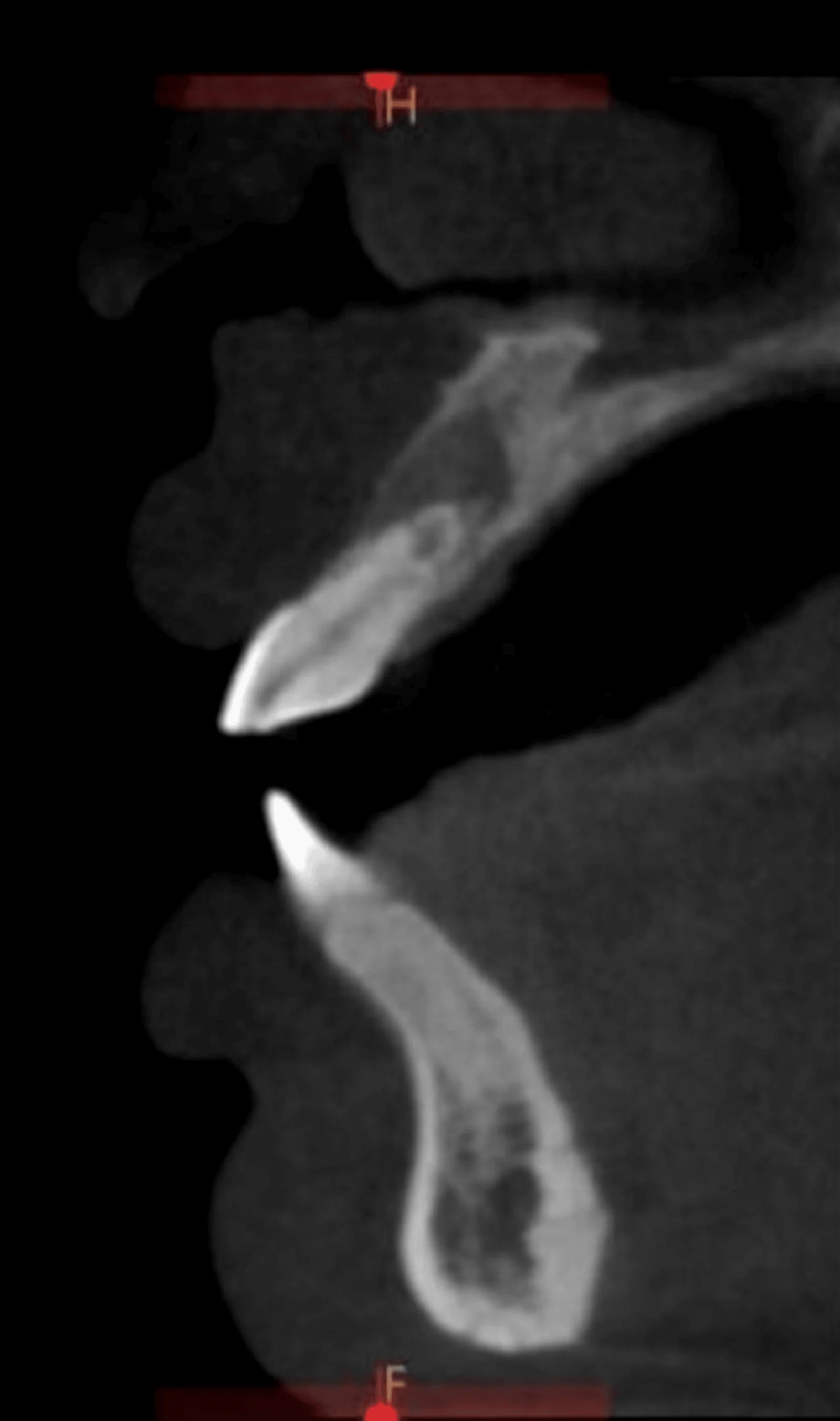
A periapical lesion at the apex of a non-endodontically treated tooth

For each ETT, the following was recorded: tooth type (incisor, premolar, or molar), root type (buccal, lingual, mesiobuccal, distobuccal, or palatal), presence or absence of periapical radiolucency (Figure 1), presence or absence of missed canals, and quality or length of the filling material.

**Figure 2:**
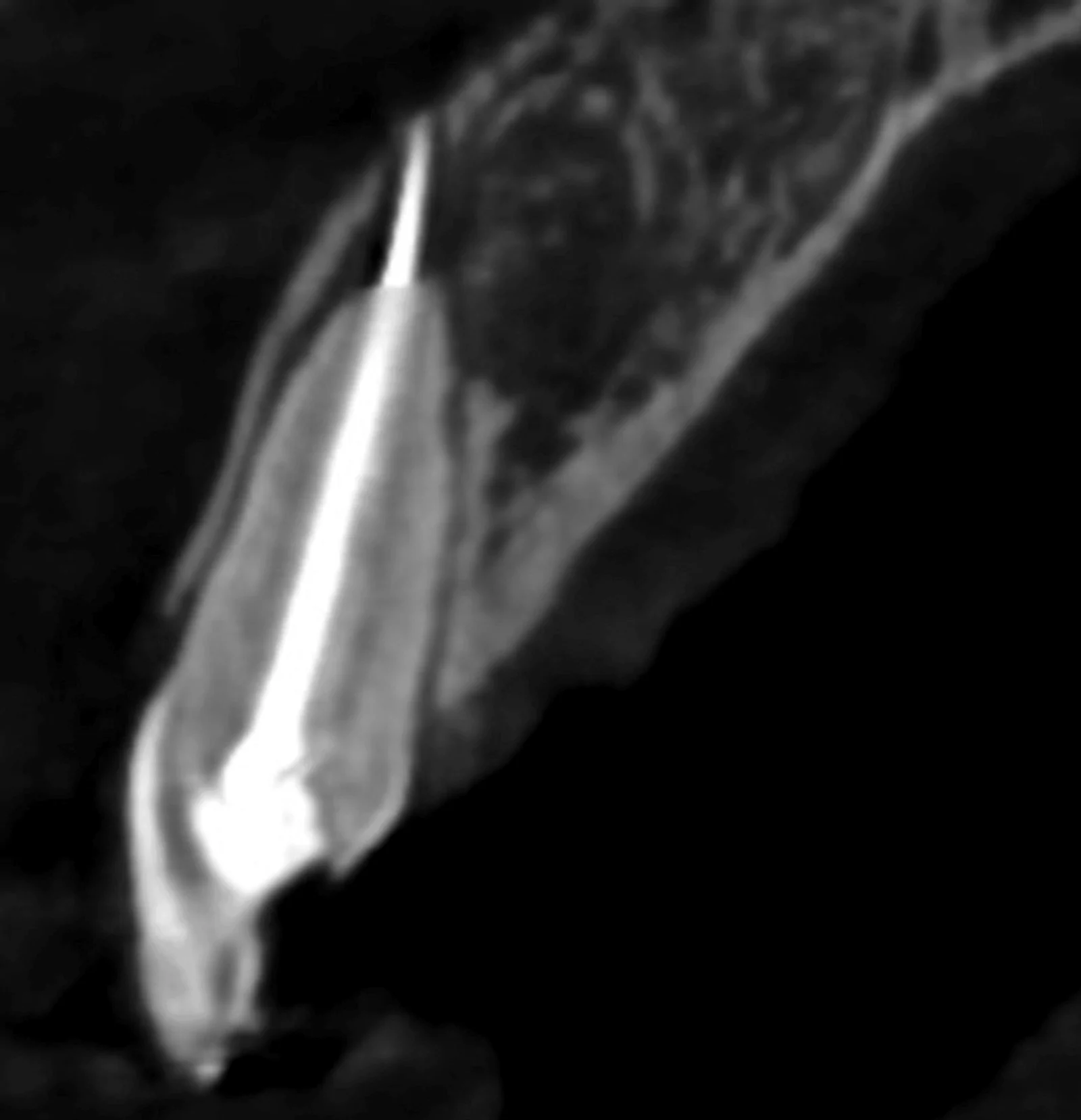
A periapical lesion at the apex of an endodontically treated tooth with an overfilling in the obturation material

Periapical lesions were identified where there is a bony socket margin that was poorly defined or irregular and accompanied by periapical hypodensity suggestive of bone resorption at the root apex. The quality of the filling material was divided into three classes: class 0 indicating a short filling (>2 mm away from the apical foramen), class 1 indicating a good filling (0-2 mm away from the apical foramen), and class 2 indicating an overfilling of the material out of the apical foramen (Figure 2).

Statistical analysis 

The analytic unit was the tooth/root record. Since multiple tooth/root observations were derived from the same patient, all inferential analyses accounted for within-patient clustering. All statistical analyses were performed using IBM SPSS Statistics for Windows, Version 26.0 (IBM Corp., Armonk, New York, United States). A significance level of α=0.05 was applied to all analyses.

Descriptive statistics were calculated at both the patient and tooth/root levels. Categorical variables were presented as frequencies and percentages. Age was summarized using mean and standard deviation (SD), median and interquartile range (IQR), and minimum and maximum values.

Inferential analyses were performed using generalized estimating equation (GEE) logistic regression models with a binomial distribution, logit link function, exchangeable working correlation matrix, and robust standard errors to account for the clustering of observations within patients. For the first study objective, an adjusted GEE model was used to evaluate the association between periapical lesions and endodontic treatment status (ETT versus non-ETT), controlling for age, gender, tooth type, and jaw location. For the second objective, analyses were restricted to ETT, and adjusted GEE models were used to assess the associations between periapical lesions and missed canal status as well as obturation length while controlling for age, gender, tooth type, and jaw location.

Additionally, a patient-level analysis was performed by creating a binary outcome variable indicating the presence of at least one periapical lesion (AnyPL) per patient. Data were aggregated to a single record per patient, and binary logistic regression was used to evaluate the associations between AnyPL and patient age and gender.

## Results

A total of 183 patients, divided between 98 females and 85 males, contributed to the study allowing 3500 tooth/root observations. The mean age was 47.4±14.63 years (range: 18-81). Eight hundred and seventy-five teeth underwent endodontic treatment, while the remaining 2625 teeth did not, with only 424 teeth presenting with a periapical lesion. For every ETT, the presence of a missed canal along with the obturation length was recorded (Table 1). 

**Table 1:** Descriptive characteristics of the study sample (patients; n=183) and tooth/root observations (n=3500) ETT-only variables (missed canal, obturation length) are calculated among ETT observations with non-missing values (coded 9 treated as not applicable). ETT: endodontically treated teeth

Variable	n	%
Gender		
Female	98	53.6
Male	85	46.4
Endodontic treatment status		
Non-ETT	2625	75
ETT	875	25
Periapical lesion		
Absent	3076	87.9
Present	424	12.1
Jaw		
Maxilla	2047	58.5
Mandible	1453	41.5
Tooth type		
Molar	1347	38.5
Incisor	924	26.4
Premolar	762	21.8
Canine	467	13.3
Missed canal (ETT only)		
No	834	94.9
Yes	45	5.1
Missed canal type (missed canal only)		
Buccal	4	8.89
Distobuccal	5	11.11
Distal	1	2.22
Lingual	9	20
Mesiobuccal 1	1	2.22
Mesiobuccal 2	16	35.56
Mesial	1	2.22
Palatal	8	17.78
Obturation length (ETT only)		
Adequate (0-2 mm)	584	66.7
Short (>2 mm)	279	31.9
Overfilled	12	1.4

The crude prevalence of periapical lesions by endodontic treatment status showed higher odds in ETT compared with non-ETT (crude OR: 13.57) (Table 2). Using a univariable GEE logistic model clustered on patient, the unadjusted association remained strong (OR: 10.74; 95% CI: 6.92-16.67; p<0.001) (Table 2). In the multivariable GEE logistic regression model adjusted for age, gender, tooth type, and jaw, ETT status remained strongly associated with periapical lesion (aOR: 8.79; 95% CI: 5.65-13.68; p<0.001). However, the jaw was not associated with periapical lesion after adjustment (aOR: 1.10; 95% CI: 0.82-1.48; p=0.526). Tooth type was independently associated with periapical lesion: compared with incisors, canines had lower odds (aOR: 0.59; 95% CI: 0.38-0.93; p=0.022), while premolars (aOR: 1.50; 95% CI: 1.01-2.24; p=0.046) and molars (aOR: 1.75; 95% CI: 1.12-2.73; p=0.015) had higher odds (Table 3).

**Table 2:** Crude prevalence of PL by endodontic treatment status and, among ETT, by missed canal status and obturation length category *Statistically significant: p<0.05 Reference categories: non-ETT (ETT status), no missed canal (missed canal status), and adequate obturation 0-2 mm (obturation length). Univariable GEE models were clustered by patient ID. PL: periapical lesion; ETT: endodontically treated teeth; GEE: generalized estimating equation

Variable	Category	PL present	Crude OR	Univariable GEE	
				OR (95% CI)	P-value
Endodontic treatment status	Non-ETT (n=2625)	106 (4%)		10.74 (6.92-16.67)	<0.001*
	ETT (n=875)	318 (36.3%)	13.57		
Missed canal status (ETT only)	No (n=830)	294 (35.4%)		1.65 (0.92-2.96)	0.092
	Yes (n=45)	24 (53.3%)	2.08		
Obturation length category	Adequate (0-2 mm) (n=584)	172 (29.5%)			
	Short (>2 mm) (n=279)	139 (49.8%)	2.38	2.62 (1.71-4.00)	<0.001*
	Overfilled (n=12)	7 (58.3%)	3.35	3.55 (1.30-9.71)	0.013*

**Table 3:** Cluster-adjusted association with PL (GEE logistic regression). Outcome: PL; cluster: patient ID; adjusted for age, gender, tooth type, and jaw *Statistically significant: p<0.05 Reference categories: non-ETT (ETT status), male (gender), incisor (tooth type), and mandibular (jaw). PL: periapical lesion; ETT: endodontically treated teeth; GEE: generalized estimating equation

Predictor	aOR	95% CI	P-value
ETT (vs. non-ETT)	8.79	5.65-13.68	<0.001*
Age (per 1 year)	1.00	0.99-1.02	0.554
Gender	0.79	0.52-1.19	0.264
Tooth type			
Canine vs. incisor	0.59	0.38-0.93	0.022*
Premolar vs. incisor	1.50	1.01-2.24	0.046*
Molar vs. incisor	1.75	1.12-2.73	0.015
Jaw: maxilla vs. mandible	1.10	0.82-1.48	0.526

Among ETT, crude periapical lesion prevalence is summarized by missed canal status and obturation length category (Table 2). Presence of a missed canal was associated with higher crude periapical lesion prevalence (53.3% vs. 35.4%); however, the univariable clustered GEE model did not show a statistically significant association (OR: 1.65; 95% CI: 0.92-2.96; p=0.092).

For obturation length, overfilled canals showed a higher crude OR value of 3.35 than the short-filled ones (crude OR: 2.38). In addition, the short-filled canals were associated with higher odds of periapical lesion in univariable clustered analysis (OR: 2.62; 95% CI: 1.71-4.00; p<0.001), and overfilled ones were also associated with higher odds (OR: 3.55; 95% CI: 1.30-9.71; p=0.013) (Table 2).

In the multivariable ETT-only GEE model, obturation length remained significantly associated with periapical lesion (short vs. adequate: aOR: 2.58; 95% CI: 1.69-3.93; p<0.001; overfill vs. adequate: aOR: 3.60; 95% CI: 1.34-9.70; p=0.011), while missed canal status was not statistically significant after adjustment (aOR: 1.61; 95% CI: 0.85-3.04; p=0.141) (Table 4). Tooth type and jaw were not significant in the ETT-only model (all p>0.05).

**Table 4:** ETT only: cluster-adjusted predictors of PL (GEE logistic regression). Outcome: PL; cluster: patient ID; adjusted for age, gender, tooth type, and jaw *Statistically significant: p<0.05 Reference categories: no missed canal (missed canal status), adequate obturation (0-2 mm) (obturation length), male (gender), incisor (tooth type), and mandibular (jaw). PL: periapical lesion; ETT: endodontically treated teeth; GEE: generalized estimating equation

Predictor	aOR	95% CI	P-value
Missed canal (yes vs. no)	1.61	0.85-3.04	0.141
Obturation length category			
Short filling vs. adequate	2.58	1.69-3.93	<0.001*
Overfill vs. adequate	3.60	1.34-9.70	0.011*
Age (per 1 year)	1.01	0.99-1.02	0.551
Gender	0.80	0.52-1.25	0.328
Tooth type			
Canine vs. incisor	1.05	0.55-2.02	0.878
Premolar vs. incisor	1.38	0.74-2.59	0.313
Molar vs. incisor	1.24	0.63-2.42	0.537
Jaw: maxilla vs. mandible	1.03	0.67-1.59	0.883

Age and gender were included as covariates in tooth/root-level GEE models and were not statistically significant after adjustment (Tables 3-4). In the supplementary patient-level analysis, 64.8% of patients had ≥1 periapical lesion (AnyPL=1; 95% CI: 57.7-71.4%). Patient-level logistic regression showed a small, non-significant increase in odds per year of age (OR: 1.02; 95% CI: 1.00-1.04; p=0.100) and no significant association with gender (OR: 1.05; 95% CI: 0.57-1.95; p=0.866; Table 5).

**Table 5:** Patient-level associations of age and gender with AnyPL (≥1 periapical lesion per patient): binary logistic regression (n=183) *Statistically significant: p<0.05 Reference category: male

Predictor	OR	95% CI	P-value
Age (per 1 year)	1.02	1.00-1.04	0.100
Gender	1.05	0.57-1.95	0.866

## Discussion

This study was performed to evaluate the presence of periapical lesions in ETT, along with its association with the presence of missed canals and the root canal filling length independently, in a Lebanese subpopulation using CBCT. The success of endodontic treatment is commonly assessed by the absence of periapical radiolucency on radiographic examination [16]. However, failed endodontic treatment may exhibit a broad spectrum of clinical outcomes, from asymptomatic cases with no detectable signs and symptoms to severe manifestations such as acute apical abscesses [11,17]. Therefore, apical periodontitis develops as a consequence of bacterial infection within the root canal system, leading to inflammation of the periapical tissues [2,4].

In comparison to previous studies, the presence of a periapical lesion showed a similar prevalence in the Lebanese subpopulation, 424 (12.1%), with respect to other populations [5,17-20]. However, these studies were based on panoramic and periapical radiographs, whereas the present study utilized CBCT. The superior diagnostic sensitivity of CBCT may explain the higher prevalence of apical periodontitis observed in the current study. Consistent with this explanation, Estrela et al. demonstrated that apical periodontitis was identified in 17.6% of ETT using panoramic radiographs, 35.3% using periapical radiographs, and 63.3% using CBCT [21]. The majority of these lesions (318 (36.3%)) were present in ETT. Nevertheless, the present results are in agreement with those of other studies conducted in various populations, indicating that the presence of a root canal filling significantly increases the likelihood of radiographically detecting apical periodontitis [18,22]. It should be noted that molars and premolars showed higher prevalence than anterior teeth.

Identifying missed canals remains a challenge, as no specific clinical signs or symptoms can be exclusively attributed to their presence. Consequently, in ETT, missed canals are considered a clinically significant finding, with studies focusing on their prevalence and their association with the development of apical periodontitis [17,23]. Out of 875 ETT, only 45 (5.1%) presented with missed canals, which is a smaller percentage compared to other studies which were mostly around 12% [17,23]. Some studies even showed higher results of 38%, mainly due to the inclusion of only premolars and molars [24].

The most missed canal to be treated is the second mesiobuccal canal (n=16; 35.56%), which is consistent with Baruwa et al. [17] and Ebrahimi et al. [11]. In this subpopulation, we are closer to the values shown in the Indian and Pakistani populations (29.4% and 21.52%, respectively) [25,26]. However, other studies showed a higher prevalence of 87%, like in Jordan [25]. These high values are due to the complex and highly variable morphology of the mesiobuccal root, which can increase the likelihood of canals being overlooked during endodontic treatment [27,28]. Missed canals may act as reservoirs for persistent microorganisms within previously infected root canal systems, particularly in roots with Vertucci type IV and V configurations, where two separate canals extend to two independent apical foramina. Since these configurations have been reported in approximately 42.7% of cases, failure to treat one of these canals may permit bacterial persistence and apical migration, maintaining communication with the periapical tissues [17,29]. This anatomical feature may account for the high prevalence of periapical lesions observed in association with mesiobuccal roots in the present study.

In the present study, the prevalence of periapical lesions in ETT with missed canals is 24 (53.3%), with a p-value of 0.092, showing no significance, in contrast to other studies where an odds ratio of 4.4 was easily noticeable [17,23]. This indicates that the presence of missed canals does increase the prevalence of periapical lesions in the Lebanese subpopulation but not as significantly as in the rest of the populations.

The optimal length of root canal obturation remains controversial [15]. Numerous studies have reported higher success rates and a lower prevalence of apical periodontitis when the obturation terminates 1-2 mm short of the radiographic apex, largely due to the preservation of periapical tissues and prevention of material extrusion [13,15,30]. Conversely, more recent recommendations support obturation up to the apical terminus, particularly in necrotic teeth, to achieve the optimal sealing of the root canal system [8]. The widespread adoption of thermoplastic obturation techniques has further contributed to the frequency of fillings reaching the apex or exhibiting sealer extrusion beyond it, highlighting the ongoing debate regarding the ideal obturation length [31]. Most canals presented in this study fell into the adequate filling category (0-2 mm) (n=584; 66.7%), followed by short filling (n=279; 31.9%), and very few presented with overfilling (n=12; 1.4%).

Despite the few overfilled canals (n=7), 58.3% were associated with periapical lesions, with a significant p-value of 0.013. This is higher than Liang et al. [14] and Byström et al. [32] with 33% and 38%, respectively. In nearly 80% of roots, the apical foramen does not coincide with the anatomical apex and may be situated as much as 3.8 mm coronally, often on the buccal or lingual aspect of the root [33]. This anatomical variation may explain the tendency for instruments and obturation materials to extend beyond the apical foramen even when confined to within 2 mm of the radiographic apex on periapical radiographs [34,35].

Since the purpose of obturation is to create a hermetic seal that prevents the proliferation of residual bacteria remaining after mechanical and chemical debridement, inadequate filling of the main canal may facilitate microbial persistence and lead to a higher prevalence of apical periodontitis [36]. Short canals presented with a 49.8% association with periapical lesions with a significant p-value of 0.001. This showed a lower prevalence than the overextension in canals in contrast to other studies where the higher association was in short-filled canals [5,37,38].

In the present study, neither age nor gender had any influence on the prevalence of periapical lesion and its association with missed canals or the quality of the root fillings.

Advances in CBCT technology have significantly improved the radiographic diagnosis of endodontic pathology [39-41]. Unlike two-dimensional imaging, which may fail to reveal periapical lesions until substantial 30-50% mineralized bone loss has occurred, CBCT offers three-dimensional visualization that facilitates the detection of early periapical changes [21,42]. Previous studies have reported greater diagnostic accuracy with CBCT, including a 38% increase in the identification of periapical lesions compared with conventional radiographs [43]. This enhanced capability allows for more precise assessment of lesion extent, bone loss, and prognosis, thereby contributing to better-informed clinical decision-making. However, despite its diagnostic advantages, clinicians should remain aware of the possibility of overestimating the presence of apical periodontitis when interpreting CBCT images [44].

Limitations

The cross-sectional nature of this study restricted the assessment to a single time point, without considering the duration since the completion of endodontic treatment. As a result, changes in the size or healing status of periapical lesions over time could not be evaluated. No clinical symptoms, periodontal health, or coronal restoration of the endodontic teeth were taken into consideration. Furthermore, the absence of microbiological and histological analyses prevented the definitive confirmation of the relationship between missed or untreated canals and periapical pathology. It should also be noted that all the CBCTs used were from the same radiological center and only cover a subpopulation of Lebanon.

## Conclusions

The CBCT-based cross-sectional study showed a high prevalence of periapical lesions in ETT. It also found that ETT and inadequate obturation length were associated with a higher prevalence of periapical lesions. Missed canals showed a higher crude prevalence of lesions, but this association was not statistically significant after adjustment. Therefore, knowledge of the anatomy and morphology of each tooth and the correct use of instrumentation and filling materials is crucial for the success of endodontic treatment. This requires constant learning and advancements in daily practice. Further longitudinal studies are needed to confirm these findings.

## References

[1] Ricucci D, Siqueira JF Jr (2010). Biofilms and apical periodontitis: study of prevalence and association with clinical and histopathologic findings. J Endod.

[2] Hou Y, Wang L, Zhang L, Tan X, Huang D, Song D (2022). Potential relationship between clinical symptoms and the root canal microbiomes of root filled teeth based on the next-generation sequencing. Int Endod J.

[3] (2019). Essential Endodontology: Prevention and Treatment of Apical Periodontitis.

[4] Siqueira JF Jr, Rôças IN (2022). Present status and future directions: microbiology of endodontic infections. Int Endod J.

[5] Covello F, Franco V, Schiavetti R, Clementini M, Mannocci A, Ottria L, Costacurta M (2010). Prevalence of apical periodontitis and quality of endodontic treatment in an Italian adult population. Oral Implantol (Rome).

[6] Kassebaum NJ, Bernabé E, Dahiya M, Bhandari B, Murray CJ, Marcenes W (2015). Global burden of untreated caries: a systematic review and metaregression. J Dent Res.

[7] El Ouarti I, Chala S, Sakout M, Abdallaoui F (2021). Prevalence and risk factors of apical periodontitis in endodontically treated teeth: cross-sectional study in an adult Moroccan subpopulation. BMC Oral Health.

[8] Chugal NM, Clive JM, Spångberg LS (2003). Endodontic infection: some biologic and treatment factors associated with outcome. Oral Surg Oral Med Oral Pathol Oral Radiol Endod.

[9] Mashyakhy M, Hadi FA, Alhazmi HA (2021). Prevalence of missed canals and their association with apical periodontitis in posterior endodontically treated teeth: a CBCT study. Int J Dent.

[10] León-López M, Montero-Miralles P, Cabanillas-Balsera D, Saúco-Márquez JJ, Martín-González J, Segura-Egea JJ (2025). Association between the presence of missed canals, detected using CBCT, and post-treatment apical periodontitis in root-filled teeth: a systematic review and meta-analysis. J Clin Med.

[11] Ebrahimi R, Khajeh S, Paik H, Moradi M, Rastegar Khosravi M (2024). Prevalence of untreated canals and their association with periapical periodontitis using cone-beam computed tomography. Iran Endod J.

[12] Hao J, Liu H, Shen Y (2023). Periapical lesions and missed canals in endodontically treated teeth: a cone-beam computed tomographic study of a Chinese subpopulation. Med Sci Monit.

[13] de Sousa Gomide Guimarães MR, Samuel RO, Guimarães G, Nalin EK, Bernardo RT, Dezan-Júnior E, Cintra LT (2019). Evaluation of the relationship between obturation length and presence of apical periodontitis by CBCT: an observational cross-sectional study. Clin Oral Investig.

[14] Liang YH, Li G, Shemesh H, Wesselink PR, Wu MK (2012). The association between complete absence of post-treatment periapical lesion and quality of root canal filling. Clin Oral Investig.

[15] Moura MS, Guedes OA, De Alencar AH, Azevedo BC, Estrela C (2009). Influence of length of root canal obturation on apical periodontitis detected by periapical radiography and cone beam computed tomography. J Endod.

[16] Bender IB, Seltzer S, Soltanoff W (1966). Endodontic success-a reappraisal of criteria: part II. Oral Surg Oral Med Oral Pathol.

[17] Baruwa AO, Martins JN, Meirinhos J (2020). The influence of missed canals on the prevalence of periapical lesions in endodontically treated teeth: a cross-sectional study. J Endod.

[18] Ali AH, Mahdee AF, Fadhil NH, Shihab DM (2022). Prevalence of periapical lesions in non-endodontically and endodontically treated teeth in an urban Iraqi adult subpopulation: a retrospective CBCT analysis. J Clin Exp Dent.

[19] Tibúrcio-Machado CS, Michelon C, Zanatta FB, Gomes MS, Marin JA, Bier CA (2021). The global prevalence of apical periodontitis: a systematic review and meta-analysis. Int Endod J.

[20] Gulsahi K, Gulsahi A, Ungor M, Genc Y (2008). Frequency of root-filled teeth and prevalence of apical periodontitis in an adult Turkish population. Int Endod J.

[21] Estrela C, Bueno MR, Leles CR, Azevedo B, Azevedo JR (2008). Accuracy of cone beam computed tomography and panoramic and periapical radiography for detection of apical periodontitis. J Endod.

[22] Bürklein S, Schäfer E, Jöhren HP, Donnermeyer D (2020). Quality of root canal fillings and prevalence of apical radiolucencies in a German population: a CBCT analysis. Clin Oral Investig.

[23] Costa FF, Pacheco-Yanes J, Siqueira JF Jr (2019). Association between missed canals and apical periodontitis. Int Endod J.

[24] Karabucak B, Bunes A, Chehoud C, Kohli MR, Setzer F (2016). Prevalence of apical periodontitis in endodontically treated premolars and molars with untreated canal: a cone-beam computed tomography study. J Endod.

[25] Alotaibi BB, Khan KI, Javed MQ, Dutta SD, Shaikh SS, Almutairi NM (2024). Relationship between apical periodontitis and missed canals in mesio-buccal roots of maxillary molars: CBCT study. J Taibah Univ Med Sci.

[26] Shetty H, Sontakke S, Karjodkar F, Gupta P, Mandwe A, Banga KS (2017). A cone beam computed tomography (CBCT) evaluation of MB2 canals in endodontically treated permanent maxillary molars. A retrospective study in Indian population. J Clin Exp Dent.

[27] Yoshioka T, Kikuchi I, Fukumoto Y, Kobayashi C, Suda H (2005). Detection of the second mesiobuccal canal in mesiobuccal roots of maxillary molar teeth ex vivo. Int Endod J.

[28] von Arx T (2005). Frequency and type of canal isthmuses in first molars detected by endoscopic inspection during periradicular surgery. Int Endod J.

[29] Kim Y, Lee SJ, Woo J (2012). Morphology of maxillary first and second molars analyzed by cone-beam computed tomography in a Korean population: variations in the number of roots and canals and the incidence of fusion. J Endod.

[30] Holland R, Gomes JE Filho, Cintra LT, Queiroz ÍOA, Estrela C (2017). Factors affecting the periapical healing process of endodontically treated teeth. J Appl Oral Sci.

[31] Heeren TJ, Levitan ME (2012). Effect of canal preparation on fill length in straight root canals obturated with RealSeal 1 and Thermafil Plus. J Endod.

[32] Bystrom A, Happonen RP, Sjogren U, Sundqvist G (1987). Healing of periapical lesions of pulpless teeth after endodontic treatment with controlled asepsis. Endod Dent Traumatol.

[33] Dummer PM, McGinn JH, Rees DG (1984). The position and topography of the apical canal constriction and apical foramen. Int Endod J.

[34] Stein TJ, Corcoran JF (1992). Radiographic "working length" revisited. Oral Surg Oral Med Oral Pathol.

[35] ElAyouti A, Weiger R, Löst C (2001). Frequency of overinstrumentation with an acceptable radiographic working length. J Endod.

[36] Endal U, Shen Y, Knut A, Gao Y, Haapasalo M (2011). A high-resolution computed tomographic study of changes in root canal isthmus area by instrumentation and root filling. J Endod.

[37] Kabak Y, Abbott PV (2005). Prevalence of apical periodontitis and the quality of endodontic treatment in an adult Belarusian population. Int Endod J.

[38] De Moor RJ, Hommez GM, De Boever JG, Delmé KI, Martens GE (2000). Periapical health related to the quality of root canal treatment in a Belgian population. Int Endod J.

[39] Patel S, Dawood A, Mannocci F, Wilson R, Pitt Ford T (2009). Detection of periapical bone defects in human jaws using cone beam computed tomography and intraoral radiography. Int Endod J.

[40] Weissman J, Johnson JD, Anderson M (2015). Association between the presence of apical periodontitis and clinical symptoms in endodontic patients using cone-beam computed tomography and periapical radiographs. J Endod.

[41] Venskutonis T, Plotino G, Tocci L, Gambarini G, Maminskas J, Juodzbalys G (2015). Periapical and endodontic status scale based on periapical bone lesions and endodontic treatment quality evaluation using cone-beam computed tomography. J Endod.

[42] de Paula-Silva FW, Wu MK, Leonardo MR, da Silva LA, Wesselink PR (2009). Accuracy of periapical radiography and cone-beam computed tomography scans in diagnosing apical periodontitis using histopathological findings as a gold standard. J Endod.

[43] Lofthag-Hansen S, Huumonen S, Gröndahl K, Gröndahl HG (2007). Limited cone-beam CT and intraoral radiography for the diagnosis of periapical pathology. Oral Surg Oral Med Oral Pathol Oral Radiol Endod.

[44] Kruse C, Spin-Neto R, Reibel J, Wenzel A, Kirkevang LL (2017). Diagnostic validity of periapical radiography and CBCT for assessing periapical lesions that persist after endodontic surgery. Dentomaxillofac Radiol.

